# 

**Published:** 1883-05

**Authors:** 


ORIGINAL COMMUNICATIONS:

Abscesses of the Ano-Perineal Region.—Philip S. Wales, M. D...... 249
Dental Caries.—Dr. W. D. Miller.................................. 260

SELECTIONS:

The Vegetable Nature of Croup.................................... 270

EDITORIAL:

To Young Practitioners........................................... 277
The New Medical Code............................................. 282
A Look Ahead..................................................... 283
An Apology....................................................... 284
Dr. Leigh H. Hunt................................................ 284
Hardening Plaster Casts.......................................... 285
Our Exchanges.................................................... 285

CURRENT NEWS AND OPINION:

Southern Dental Association and Georgia State Dental Society....... 286
The Missouri Dental Journal...................................... 286
Union Meeting at Springfield, Mass............................... 287
Complimentary.................................................... 287

BIBLIOGRAPHICAL:

A Handbook of Materia-Medica and Theraputics for Dentists and Dental Students...... 288
Some of the Opportunities, Responsibilities, and Encouragements of Life...... 289

ORIGINAL TRANSLATIONS AND ABSTRACTS:

Dental Society of the State of New York.......................... 289
Hemorrhage after Tooth Extraction................................ 292
Gold Foil in Germany............................................. 293
Expensive Treatment.............................................. 294

POPULAR SCIENCE DEPARTMENT:

Light and Heat................................................... 294
Tides in Inland Waters........................................... 295
Absorption of Water by Plants.................................... 295
Restoring Color in Woolen or Cotton Fabrics...................... 295
Eating before Sleeping........................................... 295
Soldering Coarse Metals.......................................... 296
Another Test for Albumen in Urine................................ 297
Artificial Incubation for Infants................................. 298

